# Commentary: Emergence of a Stable Cortical Map for Neuroprosthetic Control

**DOI:** 10.3389/fnins.2017.00642

**Published:** 2017-11-24

**Authors:** Mikhail A. Lebedev

**Affiliations:** Department of Neurobiology, Duke University, Durham, NC, United States

**Keywords:** brain-machine interfaces, plasticity, linear models, fixed decoder, preferred direction

This highly cited paper by Ganguly and Carmena (2009) reported a case of neuroplasticity associated with the operation of a brain-machine interface (BMI). Neuroplasticity is of great interest to BMI developers because of its causal role in the embodiment of neural prostheses (Lebedev and Nicolelis, 2006; Dobkin, 2007; Koralek et al., 2012; Shenoy and Carmena, 2014; Kraus et al., 2016; Gulati et al., 2017).

Ganguly and Carmena reported that small populations of neurons (from 10 to 15) recorded in monkey primary motor cortex (M1) adapted to operating a BMI based on a fixed linear decoder. The decoder was trained once and left unchanged for several weeks. The population activity patterns underwent plastic modifications and stabilized on an optimal “cortical map” that assured accurate performance of center-out movements with a screen cursor. Moreover, monkeys learned to operate shuffled decoders, where the original neuronal weights were randomly reassigned.

Here I comment on three issues arising from this paper: (1) the proper way to assess neuronal tuning under BMI control; (2) the constraints imposed on neuronal tuning properties by a fixed decoder; and (3) the problem of measuring changes in tuning when both neuronal activity and cursor trajectories change.

## Neuronal tuning under BMI control

Classically, neuronal directional tuning is the dependence of neuronal firing rate on the direction of arm reaching movement (Georgopoulos et al., 1982; Schwartz et al., 1988). Typically, an M1 neuron exhibits the highest firing rate when the arm moves in the direction, called preferred direction (PD). If the arm moves at an angle with respect to the PD, firing rate declines proportionally to the cosine of the angle.

While it is tempting to use a directional tuning analysis for BMI control (Taylor et al., 2002; Lebedev et al., 2005; Ganguly and Carmena, 2009), the results of such an analysis should be interpreted with caution. The main caveat here (and this is rarely explained in the literature) is the strong dependence of neuronal tuning characteristics on the decoder parameters. Taking a single-tap positional decoder (Georgopoulos et al., 1983, 1986; Taylor et al., 2002; Schwartz et al., 2004) as an example, the relationship between the firing rate of a given neuron, *N(t)*, and cursor coordinates, *x* and *y*, is expressed by the equations:

(1) $$\begin{array}{l} x=aN(t)+\text{contribution}\_\text{of}\_\text{other}\_\text{neurons}\\ y=bN(t)+\text{contribution}\_\text{of}\_\text{other}\_\text{neurons} \end{array}$$

Let's assume first that the correlation is very low between *N(t)* and the activity of other neurons. In this case, contribution_of_other_neurons does not interfere with the neuronal directional tuning, and the vector [*a, b*] defines the neuron's PD. This PD would persist during BMI control even if the neuron produces nonsensical firing unrelated to motor commands or feedback from the cursor.

Next, if the correlation of *N(t)* with the activity of the other neurons is substantial, the neuron's PD may be different from [*a, b*]. Consider the case of two neurons with positively correlated rates:

(2) $$\begin{array}{l} x=a_{1}N1(t)+a_{2}N2(t)\\ y=b_{1}N1(t)+b_{2}N2(t)\\ N2(t)=KN1(t)+\text{noise} \end{array}$$

In this case, the PDs of neurons 1 and 2 are [*a*_1_ + *Ka*_2_, *b*_1_ + *Kb*_2_] and [*a*_1_/*K* + *a*_2_, *b*_1_/*K* + *b*_2_], respectively, i.e., neurons affect each other's directional tuning. Again, these PDs would be produced even if the neuronal firing is nonsensical. Furthermore, if the contribution of one neuron, for example neuron 2, is much stronger than the contribution of the other, both have the same PD, [*a*_2_, *b*_2_]. Such “capture” of the PD by the stronger weighted neurons may explain the previously reported similarity of many neurons' PDs during BMI control (Carmena et al., 2003; Lebedev et al., 2005; Green and Kalaska, 2011; O'Doherty et al., 2011).

**Figure 1:**
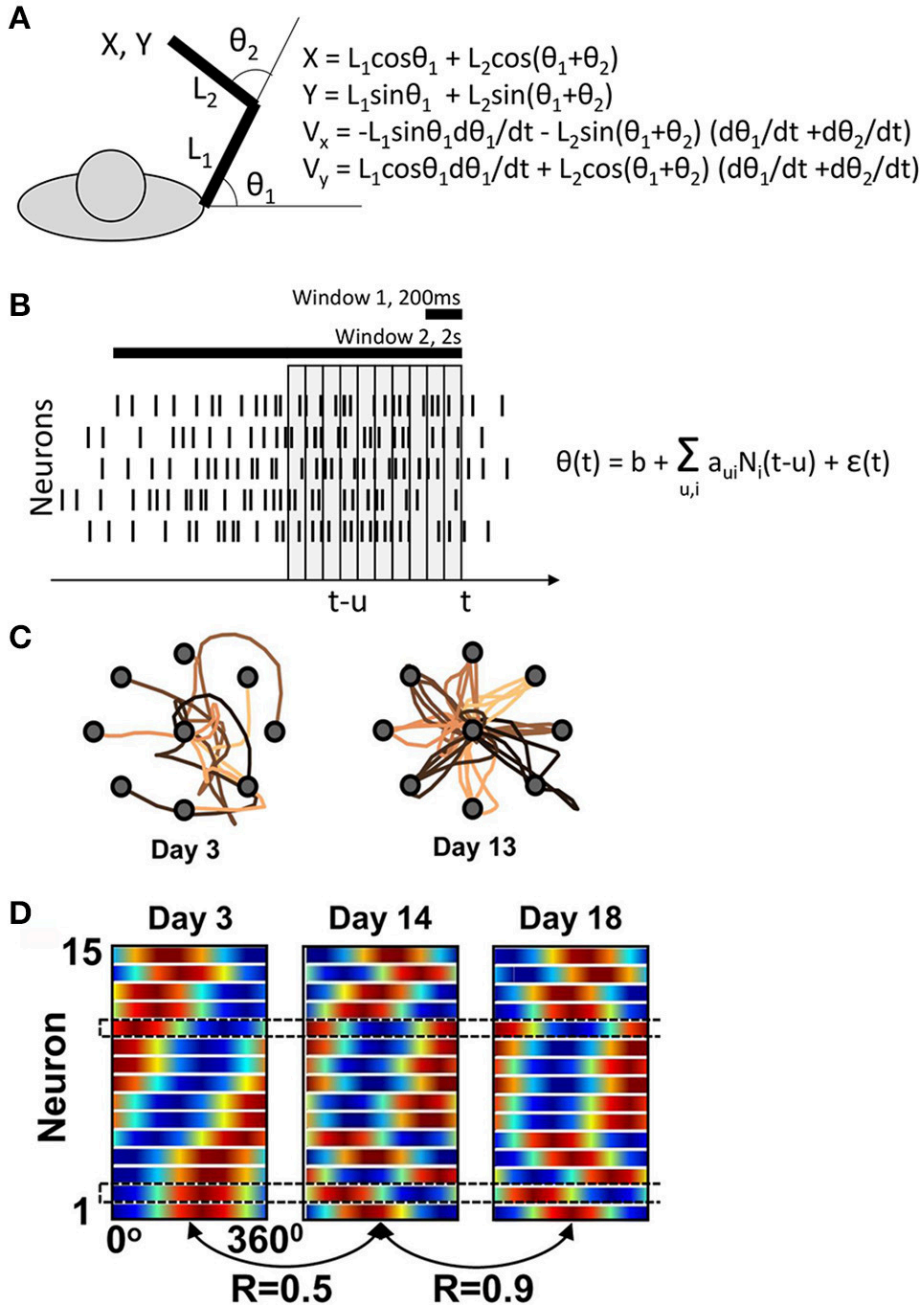
Ganguly and Carmena's experiment. **(A)** Task schematics. The behavioral apparatus suspended the arm in a horizontal plain. Monkeys were free to flex and extend their shoulder and elbow to control the cursor coordinates, defined by the center of the hand. On the right, the equations are shown for the translation of joint angles into the cursor Cartesian coordinates. **(B)** Schematics of the BMI decoder, which was composed of two Wiener filters converting neuronal activity into the shoulder and elbow angles. The decoders contained ten 100-ms taps and were trained on the manual performance in a center-out task. For BMI control, the monkeys' arms were isometrically restrained. Horizontal bars illustrate the time windows (200 ms and 2s) that Ganguly and Carmena used in their directional tuning analyses. None of these analyses matched the tap structure of the decoder. **(C)** Examples of cursor trajectories on different training days. **(D)** Directional tuning analysis using a 200-ms window representing the initial portion of cursor movement from the screen center to the target. Directional tuning characteristics for different neurons (horizontal lines) are represented by sinusoids fitted to neuronal data. The color plots are normalized to have the same minimal and maximal values for all neurons. With this representation, tuning depths cannot be compared across neurons or different training days. **(C)** and **(D)** are adapted from Ganguly and Carmena (2009).

These simple considerations are relevant to the previous studies that reported changes in PD during BMI control (Taylor et al., 2002; Green and Kalaska, 2011), including the fixed-decoder study of Ganguly and Carmena, where a linear decoder generated cursor position from the activity of a small population of M1 neurons. Ganguly's and Carmena's decoder extracted joint angles instead of *x* and *y* coordinates (Figures 1A,B), but for simplicity a linear approximation can be used:

(3) $$\begin{array}{l} X\left(t\right)=b+\sum_{u,i}a_{ui}N_{i}(t-u) \end{array}$$

where *X(t)* is the BMI output (*x, y* and/or their time derivatives), *b* is zero intercept, *i* is neuron number, *u* is Wiener filter tap, *a*_*u*__i_ are regression weights, and *N*_*i*_ are neuronal rates. This equation differs from Equation (1) by the presence of tap structure in the representation of neuronal rates. Ganguly and Carmena used ten 100-ms taps.

Ganguly and Carmena assessed directional tuning by measuring neuronal rates in two windows: a short, 200-ms, window, and a long, 2s, window. The former was used “for the analysis of the directional modulation of the firing rate with respect to the actual direction of cursor movements”^1^ and the latter for “for calculating the mean firing rate versus target direction”. None of these analyses was designed to capture the 10-tap structure of the decoder. Furthermore, Ganguly and Carmena did not report the values of *a*_*ui*_, so at this point it is impossible to assess the PDs incorporated in the decoder.

Although Ganguly and Carmena did not analyze PD for each tap, one previous study (Lebedev et al., 2005) conducted such an analysis for three conditions: manual cursor control with a joystick, BMI control assisted by the joystick, and BMI control without arm movements. It was found that neuronal PDs rotated with incrementing taps for the first two conditions but not for the third.

## Can neuronal tuning change for a fixed decoder?

The main claim of Ganguly and Carmena is that M1 ensemble starts with one pattern of directional tuning, and then gradually adapts to a new, more stable pattern as the monkey perfects the BMI control (Figure 1D). Similar changes in PD had been previously reported by Taylor and her colleagues for an adaptive decoder (Taylor et al., 2002). But is this even possible if the decoder is fixed and its weights define a basic PD structure? The answer is “yes” because, as explained above, changes in correlation between the neurons (Equation 2) could result in PD modifications. Drifts in neuronal firing rates could explain PD changes, as well. According to Ganguly and Carmena, mean rate increased in 8 of 15 neurons in one monkey, and 6 of 10 in the other. These changes in mean rates could reconfigure the joint angles (Figure 1A), which could in turn affect the neuronal PDs measured with respect to linear displacements (Equation 3).

## Assessment of changes in neuronal tuning when cursor patterns change

While the monkeys in the experiments of Ganguly and Carmena clearly improved their performance (Figure 1C), an overt strategy (e.g., pressing on the arm restraint in different directions) cannot be ruled out. The authors reported that they “concurrently performed video and surface electromyogram (EMG) recordings from proximal muscle groups” but did not present any results that would convince that directionally tuned EMG modulations did not occur. The same authors reported a paradigm with a better control for overt strategies (Ganguly et al., 2011), but the task was different; monkeys possibly used directionally tuned preparatory activity previously reported for instructed-delay tasks (Weinrich and Wise, 1982) to drive the cursor in that study.

With or without an overt strategy, cursor trajectories changed very dramatically from the very convoluted ones during the initial training days to nearly straight lines during late in training (Figure 1C). The presence of such dramatic changes makes inadequate the analysis of PDs using the wide 2-s window that covered a convoluted trajectory in the beginning of the training and a straight trajectory in the end. The 200-ms window analysis does not appear adequate either. Indeed, cursor velocity was generated from ten 100-ms taps (Equation 3, Figure 1B). The 200-ms window represents only 20% of this neuronal contribution and also does not capture the complexity of rate changes with different taps. Clearly, a much more complex procedure was used to generate cursor movements from neuronal activity compared to the attempt to measure how the generated movements depended on the activity of individual neurons.

There is no easy solution for assessing neuronal tuning in a reliable way when cursor patterns dramatically change, but several analyses could be helpful. First, PDs could be measured for each tap individually (Lebedev et al., 2005) and compared with the tap-dependent structure of PDs (also called impulse response function) enforced by the decoder. This analysis may show a pattern of PD rotations like the one observed during manual control, meaning that the monkey learned to generate neuronal patterns matching the decoder design. Alternatively, PDs would stay the same across the taps, and this would indicate that the monkey cannot produce neuronal patterns matching the training data and uses some other strategy. The tap-dependent PD patterns could be then compared to the corresponding shapes of cursor trajectories for different training days. Additionally, since correlated neuronal activity can affect PDs during BMI control (Equation 2), changes in correlation between the neurons should be assessed, as well. Finally, a simpler one-tap decoder could be used to minimize the number of factors affecting neuronal PDs. Most importantly, PD should be treated as a parameter highly dependent on the decoder settings (Equations 1, 2) rather than a separate property of brain activity. Without such analyses, the claim that a monkey learned a fixed decoder would not be sufficiently substantiated.

## Conclusions

Ganguly and Carmena's study is very convincing regarding monkey ability to learn new types of BMI control with a fixed decoder, but less convincing regarding the neuronal mechanisms underlying this learning. Some of the questionable issues could be resolved using data analyses that match more closely the decoder structure. Yet, more experiments or analysis of previously collected data may be needed with easily tractable BMI algorithms [e.g., liner decoder with just one tap (Taylor et al., 2002)] to clarify neuronal adaptations under constraints imposed by a fixed decoder.

Since the publication of Ganguly's and Carmena's work, a dynamical-systems perspective (as opposed to representation perspective) gained popularity (Shenoy et al., 2013). This new view emphasizes changes in neuronal patterns in a multi-dimensional neuronal space and downplays the importance of the description in terms of neuronal-tuning parameters, such as PD. Among the findings that emerged from this approach is the discovery of neuronal subspaces that correspond to different types of neuronal processing (Kaufman et al., 2014; Lebedev, 2017). Moreover, it has been demonstrated that plastic adaptations related to BMI operations occur more readily if the BMI control signal is derived from particular, action-potent subspaces (Sadtler et al., 2014), the result that can be interpreted as a constraint on Ganguly's and Carmena's adaptive “cortical maps”. Yet, even with these new developments neuronal plasticity during BMI control remains a largely unexplored problem that needs proper methods for quantification. Particularly, “cortical maps” should be distinguished from the “map” applied by the decoder.

## Author contributions

The author confirms being the sole contributor of this work and approved it for publication.

### Conflict of interest statement

The author declares that the research was conducted in the absence of any commercial or financial relationships that could be construed as a potential conflict of interest.

## Footnotes

^1^The description of the 200-ms window analysis is not entirely clear in Ganguly and Carmena. To the best of my understanding, only the first 200 ms following movement onset were analyzed, and neuronal activity during this period was compared to the actual direction of cursor movement, not target direction.
